# 4-Iodo-2-methyl­aniline

**DOI:** 10.1107/S1600536808004145

**Published:** 2008-02-15

**Authors:** Wei Luo, Rui Liu, Yu-Hao Li, Wei Chen, Hong-Jun Zhu

**Affiliations:** a Department of Applied Chemistry, College of Science, Nanjing University of Technology, Nanjing 210009, People’s Republic of China

## Abstract

In the mol­ecule of the title compound, C_7_H_8_IN, the methyl C, I and N atoms lie in the benzene ring plane. In the crystal structure, inter­molecular N—H⋯N hydrogen bonds link the mol­ecules in a stacked arrangement along the *a* axis.

## Related literature

For related literature, see: Kajigaeshi *et al.* (1988 ▶). For bond-length data, see: Allen *et al.* (1987 ▶).
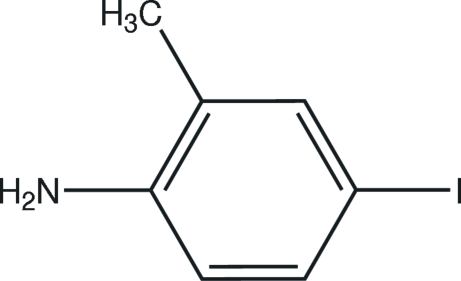


## Experimental

### 

#### Crystal data

C_7_H_8_IN
*M* *_r_* = 233.04
Orthorhombic, 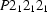

*a* = 5.5910 (11) Å
*b* = 8.9410 (18) Å
*c* = 15.674 (3) Å
*V* = 783.5 (3) Å^3^
*Z* = 4
Mo *K*α radiation
μ = 4.00 mm^−1^
*T* = 294 (2) K
0.20 × 0.10 × 0.10 mm

#### Data collection

Enraf–Nonius CAD-4 diffractometer
Absorption correction: ψ scan (North *et al.*, 1968 ▶) *T*_min_ = 0.487, *T*_max_ = 0.670
917 measured reflections
917 independent reflections
737 reflections with *I* > 2σ(*I*)
*R*_int_ = 0.012
3 standard reflections frequency: 120 min intensity decay: none

#### Refinement

*R*[*F* ^2^ > 2σ(*F* ^2^)] = 0.044
*wR*(*F* ^2^) = 0.151
*S* = 1.04
917 reflections
82 parameters
H-atom parameters constrained
Δρ_max_ = 0.51 e Å^−3^
Δρ_min_ = −0.96 e Å^−3^
Absolute structure: Flack (1983 ▶), with no Friedel pairs
Flack parameter: −0.29 (13)

### 

Data collection: *CAD-4 Software* (Enraf–Nonius, 1989 ▶); cell refinement: *CAD-4 Software*; data reduction: *XCAD4* (Harms & Wocadlo, 1995 ▶); program(s) used to solve structure: *SHELXS97* (Sheldrick, 2008 ▶); program(s) used to refine structure: *SHELXL97* (Sheldrick, 2008 ▶); molecular graphics: *PLATON-10M* (Spek, 2003 ▶); software used to prepare material for publication: *SHELXTL* (Sheldrick, 2008 ▶).

## Supplementary Material

Crystal structure: contains datablocks I, global. DOI: 10.1107/S1600536808004145/hk2418sup1.cif

Structure factors: contains datablocks I. DOI: 10.1107/S1600536808004145/hk2418Isup2.hkl

Additional supplementary materials:  crystallographic information; 3D view; checkCIF report

## Figures and Tables

**Table 1:** Hydrogen-bond geometry (Å, °)

*D*—H⋯*A*	*D*—H	H⋯*A*	*D*⋯*A*	*D*—H⋯*A*
N—H0*B*⋯N^i^	0.86	2.54	3.397 (15)	174

Symmetry code: (i) 
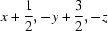
.

**Fig. 1.**
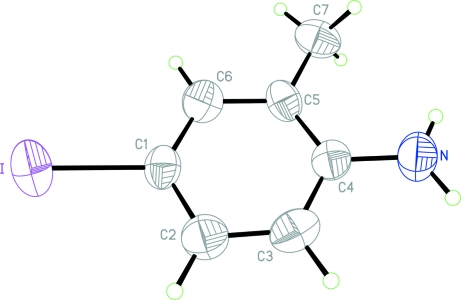
The molecular structure of the title molecule with the atom-numbering scheme. Displacement ellipsoids are drawn at the 50% probability level.

**Fig. 2.**
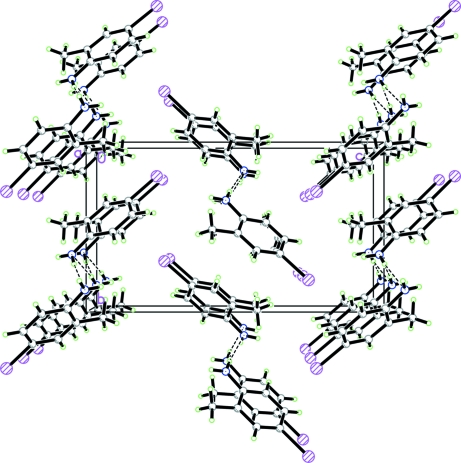
A packing diagram of (I). Hydrogen bonds are shown as dashed lines.

## supplementary crystallographic information

### Comment

The title compound, (I), contains amino and halogen groups, which can react with different groups to prepare various function organic compounds. It is a kind of aromatic organic intermediate that can be used for many fields such as aromatic conductive polymer, organometallic chemistry *etc*. (Kajigaeshi *et al.*, 1988). We report herein its crystal structure.

In the molecule of (I), (Fig. 1), the bond lengths (Allen *et al.*, 1987) and angles are within normal ranges. The atoms I, N and C7 lie in the benzene ring plane.

In the crystal structure, intermolecular N—H···N hydrogen bonds (Table 1) link the molecules stacked along the *a* axis, (Fig. 2), in which they may be effective in the stabilization of the structure.

### Experimental

The title compound, (I), was prepared by the literature method (Kajigaeshi *et al.*, 1988). Crystals suitable for X-ray analysis were obtained by dissolving (I) (0.5 g) in hexane (20 ml) and evaporating the solvent slowly at room temperature for about 7 d.

### Refinement

H atoms were positioned geometrically, with N—H = 0.86 Å (for NH_2_) and C—H = 0.93 and 0.96 Å for aromatic and methyl H, respectively, and constrained to ride on their parent atoms, with *U*_iso_(H) = *xU*_eq_(C,*N*), where *x* = 1.5 for methyl H, and *x*= 1.2 for all other H atoms.

### Crystal data

**Table:**

C_7_H_8_IN	*D*_x_ = 1.976 Mg m^−^^3^
*M**_r_* = 233.04	Melting point: 360 K
Orthorhombic, *P*2_1_2_1_2_1_	Mo *K*α radiation λ = 0.71073 Å
Hall symbol: P 2ac 2ab	Cell parameters from 25 reflections
*a* = 5.5910 (11) Å	θ = 10–14º
*b* = 8.9410 (18) Å	µ = 4.00 mm^−^^1^
*c* = 15.674 (3) Å	*T* = 294 (2) K
*V* = 783.5 (3) Å^3^	Block, purple
*Z* = 4	0.20 × 0.10 × 0.10 mm
*F*_000_ = 440	

### Data collection

**Table:**

Enraf–Nonius CAD-4 diffractometer	*R*_int_ = 0.012
Radiation source: fine-focus sealed tube	θ_max_ = 26.0º
Monochromator: graphite	θ_min_ = 2.6º
*T* = 294(2) K	*h* = 0→6
ω/2θ scans	*k* = 0→11
Absorption correction: ψ scan(North *et al.*, 1968)	*l* = 0→19
*T*_min_ = 0.487, *T*_max_ = 0.670	3 standard reflections
917 measured reflections	every 120 min
917 independent reflections	intensity decay: none
737 reflections with *I* > 2σ(*I*)	

### Refinement

**Table:**

Refinement on *F*^2^	Hydrogen site location: inferred from neighbouring sites
Least-squares matrix: full	H-atom parameters constrained
*R*[*F*^2^ > 2σ(*F*^2^)] = 0.045	*w* = 1/[σ^2^(*F*_o_^2^) + (0.1*P*)^2^ + 1.*P*] where *P* = (*F*_o_^2^ + 2*F*_c_^2^)/3
*wR*(*F*^2^) = 0.151	(Δ/σ)_max_ < 0.001
*S* = 1.04	Δρ_max_ = 0.51 e Å^−^^3^
917 reflections	Δρ_min_ = −0.96 e Å^−^^3^
82 parameters	Extinction correction: none
Primary atom site location: structure-invariant direct methods	Absolute structure: Flack (1983), with no Friedel pairs
Secondary atom site location: difference Fourier map	Flack parameter: −0.29 (13)

### Special details

**Table:**

Geometry. All e.s.d.'s (except the e.s.d. in the dihedral angle between two l.s. planes) are estimated using the full covariance matrix. The cell e.s.d.'s are taken into account individually in the estimation of e.s.d.'s in distances, angles and torsion angles; correlations between e.s.d.'s in cell parameters are only used when they are defined by crystal symmetry. An approximate (isotropic) treatment of cell e.s.d.'s is used for estimating e.s.d.'s involving l.s. planes.
Refinement. Refinement of *F*^2^ against ALL reflections. The weighted *R*-factor *wR* and goodness of fit *S* are based on *F*^2^, conventional *R*-factors *R* are based on *F*, with *F* set to zero for negative *F*^2^. The threshold expression of *F*^2^ > σ(*F*^2^) is used only for calculating *R*-factors(gt) *etc*. and is not relevant to the choice of reflections for refinement. *R*-factors based on *F*^2^ are statistically about twice as large as those based on *F*, and *R*- factors based on ALL data will be even larger.

### Fractional atomic coordinates and isotropic or equivalent isotropic displacement parameters (Å^2^)

**Table:**

	*x*	*y*	*z*	*U*_iso_*/*U*_eq_	
I	0.01067 (18)	0.20097 (9)	0.23728 (5)	0.0696 (4)	
N	0.407 (2)	0.6567 (10)	−0.0309 (8)	0.063 (3)	
H0A	0.3335	0.6711	−0.0784	0.076*	
H0B	0.5326	0.7081	−0.0188	0.076*	
C1	0.1534 (18)	0.3484 (11)	0.1464 (7)	0.044 (2)	
C2	0.360 (2)	0.4273 (12)	0.1643 (8)	0.053 (3)	
H2A	0.4425	0.4137	0.2152	0.063*	
C3	0.439 (2)	0.5276 (13)	0.1030 (7)	0.054 (3)	
H3A	0.5772	0.5825	0.1139	0.064*	
C4	0.322 (2)	0.5491 (11)	0.0269 (7)	0.042 (2)	
C5	0.122 (2)	0.4658 (11)	0.0088 (7)	0.045 (2)	
C6	0.028 (2)	0.3671 (11)	0.0701 (7)	0.051 (3)	
H6A	−0.1143	0.3158	0.0601	0.062*	
C7	−0.011 (2)	0.4852 (13)	−0.0736 (7)	0.059 (3)	
H7A	−0.1480	0.4207	−0.0741	0.089*	
H7B	0.0924	0.4599	−0.1204	0.089*	
H7C	−0.0615	0.5873	−0.0791	0.089*	

### Atomic displacement parameters (Å^2^)

**Table:**

	*U*^11^	*U*^22^	*U*^33^	*U*^12^	*U*^13^	*U*^23^
I	0.0908 (7)	0.0662 (5)	0.0517 (5)	−0.0067 (5)	0.0050 (5)	0.0104 (3)
N	0.061 (6)	0.051 (5)	0.078 (7)	−0.004 (5)	0.007 (6)	0.012 (5)
C1	0.048 (6)	0.044 (5)	0.038 (5)	0.002 (5)	0.011 (5)	−0.001 (4)
C2	0.056 (7)	0.052 (6)	0.050 (6)	−0.005 (5)	−0.005 (6)	−0.005 (5)
C3	0.041 (6)	0.055 (7)	0.064 (7)	−0.001 (5)	0.001 (5)	−0.010 (6)
C4	0.040 (6)	0.043 (6)	0.043 (5)	0.006 (4)	0.009 (5)	−0.002 (4)
C5	0.058 (7)	0.036 (5)	0.040 (5)	0.005 (5)	0.002 (5)	0.001 (4)
C6	0.056 (7)	0.044 (5)	0.054 (6)	−0.005 (6)	−0.001 (6)	−0.006 (4)
C7	0.063 (7)	0.065 (6)	0.050 (5)	0.013 (9)	−0.009 (8)	−0.006 (5)

### Geometric parameters (Å, °)

**Table:**

I—C1	2.099 (10)	C4—C5	1.374 (16)
N—H0A	0.8600	C4—N	1.403 (13)
N—H0B	0.8600	C5—C6	1.408 (15)
C1—C2	1.383 (15)	C6—H6A	0.9300
C1—C6	1.397 (15)	C7—C5	1.500 (15)
C2—C3	1.386 (16)	C7—H7A	0.9600
C2—H2A	0.9300	C7—H7B	0.9600
C3—C4	1.375 (16)	C7—H7C	0.9600
C3—H3A	0.9300		
			
C4—N—H0A	120.0	C3—C4—N	119.7 (11)
C4—N—H0B	120.0	C4—C5—C6	120.3 (10)
H0A—N—H0B	120.0	C4—C5—C7	121.2 (10)
C2—C1—C6	122.2 (10)	C6—C5—C7	118.3 (11)
C2—C1—I	120.0 (8)	C1—C6—C5	118.1 (10)
C6—C1—I	117.7 (8)	C1—C6—H6A	121.0
C1—C2—C3	117.2 (10)	C5—C6—H6A	121.0
C1—C2—H2A	121.4	C5—C7—H7A	109.5
C3—C2—H2A	121.4	C5—C7—H7B	109.5
C4—C3—C2	122.7 (10)	H7A—C7—H7B	109.5
C4—C3—H3A	118.6	C5—C7—H7C	109.5
C2—C3—H3A	118.6	H7A—C7—H7C	109.5
C5—C4—C3	119.4 (10)	H7B—C7—H7C	109.5
C5—C4—N	120.9 (11)		
			
C6—C1—C2—C3	0.2 (16)	C3—C4—C5—C6	4.6 (16)
I—C1—C2—C3	−177.3 (8)	N—C4—C5—C6	−174.8 (9)
C2—C1—C6—C5	2.5 (16)	C3—C4—C5—C7	−179.8 (10)
I—C1—C6—C5	180.0 (7)	N—C4—C5—C7	0.8 (15)
C1—C2—C3—C4	−0.5 (17)	C4—C5—C6—C1	−4.9 (16)
C2—C3—C4—C5	−1.8 (17)	C7—C5—C6—C1	179.4 (10)
C2—C3—C4—N	177.5 (10)		

### Hydrogen-bond geometry (Å, °)

**Table:**

*D*—H···*A*	*D*—H	H···*A*	*D*···*A*	*D*—H···*A*
N—H0B···N^i^	0.86	2.54	3.397 (15)	174

Symmetry codes: (i) *x*+1/2, −*y*+3/2, −*z*.

## References

[bb1] Allen, F. H., Kennard, O., Watson, D. G., Brammer, L., Orpen, A. G. & Taylor, R. (1987). *J. Chem. Soc. Perkin Trans. 2*, pp. S1–19.

[bb3] Enraf–Nonius (1989). *CAD-4 Software* Version 5.0. Enraf–Nonius, Delft, The Netherlands.

[bb4] Flack, H. D. (1983). *Acta Cryst.* A**39**, 876–881.

[bb5] Harms, K. & Wocadlo, S. (1995). *XCAD4* University of Marburg, Germany.

[bb6] Kajigaeshi, S., Kakinami, T., Yamasaki, H., Fujisaki, S. & Okamoto, T. (1988). *Bull. Chem. Soc. Jpn*, **61**, 600–602.

[bb7] North, A. C. T., Phillips, D. C. & Mathews, F. S. (1968). *Acta Cryst.* A**24**, 351–359.

[bb8] Sheldrick, G. M. (2008). *Acta Cryst.* A**64**, 112–122. 10.1107/S0108767307043930 18156677

[bb9] Spek, A. L. (2003). *J. Appl. Cryst.* **36**, 7–13.

